# Courage in Competition: Adaptation of the Sports Courage Scale for American English and Validation of the Factor Structure with Student-Athletes at Clemson University

**DOI:** 10.3390/ijerph17134834

**Published:** 2020-07-04

**Authors:** Erkut Konter, Yee Cheng Kueh, Garry Kuan

**Affiliations:** 1 Buca Educational Faculty, Dokuz Eylül University, İzmir 35380, Turkey; erkut.konter@gmail.com; 2 Unit of Biostatistics and Research Methodology, School of Medical Sciences, Universiti Sains Malaysia, Kubang Kerian 16150, Kelantan, Malaysia; 3 Exercise and Sports Science Programme, School of Health Sciences, Universiti Sains Malaysia, Kubang Kerian 16150, Kelantan, Malaysia

**Keywords:** courage scale, measurement, adaptation, sports, competitive activities

## Abstract

While courage is widely attributed to athletic pursuits, it has received little scientific attention from both researchers and applied practitioners. A reliable measurement is required to examine courage in sports and competitive activities. Therefore, this research aimed to adapt the original Turkish Sports Courage Scale-31 into American English (SCS-AE). The SCS-31 measure was translated from Turkish into the American English language by the Brislin forward and backward translation technique and language validity. Then, the translated SCS-AE was administered to 548 American university college students (Mean age = 19.02, *SD* = 1.21). All participants played a sport (e.g., football, soccer, basketball, gymnastics). Based on confirmatory factor analyses (CFA), 31 items of SCS-AE were reduced to 24 items with four factors (i.e., assertiveness, determination, mastery, and venturesome). The fit indices were satisfactory (RMSEA = 0.06, CFI = 0.97, SRMR = 0.06, NFI = 0.96 and NNFI = 0.97). The internal consistency measured by Cronbach alpha, ranging from 0.73 to 0.78, were considered acceptable. The convergent validity and discriminant validity of SCS-AE were also achieved. Our findings indicate strong support for research using the four-factor model of the SCS-AE and adequate support for the five-factor model with sufficient caution regarding the internal consistency of the self-sacrifice factor. While cultural differences in courage perception might exist between these countries, the findings showed more similarities than differences in courage. Results indicated that the SCS-AE is usable for research purposes in the suggested format. Future directions are discussed using the SCS-31 and SCS-AE for research.

## 1. Introduction

Psychological theory and practice have much to say about stress, anxiety and fear, but usually little to say about courage in general [1]. Although courage is commonly attributed to athletic pursuits, it has received little scientific attention from sport psychology researchers or applied practitioners [2,3]. Anecdotal reports suggest that courage plays an important part in individuals, teams, managers and coaches’ success, performance and health [4,5]. Courage is associated with extreme sports [6]; altruistic behavior [7]; social identity, prosocial and antisocial actions in sports for young people [8]; self-predictions including aerobic exercise [9]; and motivational antecedents of prosocial and antisocial behaviors in football [10]. While preliminary investigations are showing some promising results, an accurate and reliable measure of courage in sports is required for further research. In this study, we examine the cross-cultural validity of Konter and Ng’s [4] Sports Courage Scale, developed in Turkey and tested in the United States.

Living courageously was an important virtue in and of itself for ancient Greek philosophers, and has been shown to be deliberately sought out by participating in extreme sports that involve a real chance of death and fear [6]. However, current empirical research suggests that courageous actions are frequently instrumental in nature, only undertaken on behalf of a different, larger goal [2,11]. For example; people often describe actions with a successful outcome if they are asked to describe a courageous action [12]. Furthermore, Pury and Hensel [12] found that, while positive conduct was attenuated by external attributions, successful actions were more often regarded as courageous than unsuccessful outcomes.

Courage could be used in competitive activities and sports as an instrument for managing and overcoming anxiety, stress, pressure and fear [2]. It has been established that athletes show many aspects of courage by virtue of their basic human behavior, intellectual, cognitive, physical, emotional and social fortitude, and resolve while taking care of their opponents (sportsmanship). Furthermore, athletes who have broken their best records, taken relatively high risks and challenged their strong opponents in stressful situations, are examples of courage [1,4,13].

Courage could be importantly related to various sports psychology topics, such as self-confidence, concentration, achievement motivation, determination, competitiveness, mental toughness, hardiness, assertiveness, intelligence, creativity, coping to improve functioning and performance [4,14,15,16]. However, there are more questions than empirical answers about courage in competitive activities and sports. For instance, does courage make those aforementioned people successful in competitive activities and sports? How do those involved in competition influence their own courage and those of others to achieve their desired goals? What are the dimensions of courage in sports, and how do they relate to the dimensions of courage? Is courage a situationally-determined state or a personality trait? Are there significant differences between amateur and professional athletes, individual and team sports, on courage? To answer these questions, one needs both a better understanding of courage in competitive activities and sports and a standardized measure [3,4].

Several authors had suggested various concepts and definitions of courage [17]. Mavroudis [18] indicated that courage is the selfless pursuit of a moral good by an entity while risking personal harm, injury, or death. Next, Woodard and Pury [19] defined courage as the willingness to act willingly, with or without different degrees of fear, against a threat to achieving an important, perhaps moral, outcome or goal. Kilmann, O’Hara, and Strauss [20] examined the principles and meanings of courage, suggesting that courageous acts within organizations have five important properties: (1) Freedom of choice to decide whether to act (rather than being coerced), (2) Significant risk of being harmed, (3) The evaluation of that risk as a reasonable and contemplated act which is deemed to be justifiable (not foolhardy), (4) The pursuit of worthy aims, and (5) Proceeding with mindful actions despite fear (p. 16). Rate and colleagues [11] used implicit theory methodology and found that courage is a voluntary action taken for a good or noble goal despite the threat to the actor. These definitions recognize that fear may or may not be substantially present for an act to be considered courageous. It shows the two accepted components of courage, threats and worthy or significant outcome.

Peterson and Park [21] suggest that courage is an emotional strength, which involves a willingness to achieve goals in the face of opposition, both externally or internally, and that consists of bravery, perseverance (persistence), honesty (integrity), and zeal (vitality). Similarly, Konter [14] defined sports courage as a “natural and developed, interactional and perceptual concept between person and situation, and the task at hand that enables a person to move in competence, mastery, determination, assertiveness, venturesome and sacrificial (altruistic) behavior on a voluntary basis and in danger(ous) circumstances” (p. 966). These strengths of courage in sport should allow individuals to initiate and persist at goal pursuit despite the risk.

Konter [14] reviewed the literature and arranged individual meetings with over 60 coaches, physical education teachers and sport science academicians, and noted their interpretations, opinions and examples of courageous conduct. Then, several discussions with students and athletes were held to understand their points of view on courage in sport. In addition, to develop a model of courage in sports, data was collected from 338 athletes with open-ended questions about courage (male = 175, female = 163, professional = 65 and amateur = 273) [14]. Discussions with coaches and open-ended questions with athletes were analyzed related to theme-building procedures including similarities and differences, interactions and multidimensionality, general and specific issues. Based on the results of these procedures, models describing feelings and experiences regarding sports courage were created [13]. Konter introduced a sports-specific courage model, highlighting the interactions between variables including situations (e.g., risk, danger, fear at present), individual differences (e.g., personality traits, athlete’s experience and knowledge), sport (e.g., individual and team, contact and non-contact), and the task at hand (e.g., taking a crucial decisive penalty kick of a soccer game or free throw in basketball). Therefore, the concept of sports courage is proposed as a complex and transformational mechanism, which changes in time due to the interactions mentioned above [1,13,18].

Konter and Ng [4] recently developed the SCS-31 consisting of 5 factors, including Determination, Mastery, Assertiveness, Venturesome, and Self-Sacrificing Behavior for 13 to 22 years old athletes. The scale demonstrated test-retest reliability based on 75 athletes’ responses. Revision of the SCS-31 for children 10 to 12 years of age was obtained using the same five factors with 28 items [3]. Both scales’ analyses (SCS-31 and RSCS-Children) have shown a 5-factor SCS structure that supported factorial validity and scale scores reliability. The only difference with Revised Sports Courage Scale for children 10 to 12 years old was three new items of the Self-Sacrifice factor that were not included in the SCS-31.

Determination is described as “a personality trait which, despite barriers and difficulties, is characterized by a tendency to move its goal forward.” This also means “decision-making, with reaching of a conclusion” [22] (p. 2002). The Determination scale of the SCS-31 contains items, i.e., *I perform to the best of my ability no matter how negative the current conditions are in my sport*, and *Even when under pressure I do not lose sight of my goals in my sport*. Next, Mastery is the second factor of SCS-31. Vealey, Hayashi, Garner-Holman, and Giacobbi [23] proposed that “Mastery, as a source of self-confidence, involves performing well, improving and achieving personal goals” (p. 58). The application of skill or achievement is known as Mastery, a term taken from the ancient French word *maistre*, in the form of a commanding superior, the Latin magister. This term is particularly used in execution of sports-related skill [24] (pp. 260–261). The Mastery of the SCS-31 subscale includes reversed items such as *My doubts regarding my abilities prevent me from succeeding in my sport*, and *I become pessimistic when faced with difficult situations in my sport*.

The third factor of the SCS-31 is Assertiveness. Assertiveness is the “use of legitimate, acceptable physical force and the expenditure of an unusually high effort to achieve an external goal, without any intention of injuring” [25] (p. 54). Exemplars of Assertiveness items on the SCS-31 are *I like to take the initiative in the face of difficulties in my sport*, and *I assert myself even when facing hazardous situations in my sport*. The fourth SCS factor is Venturesome, which includes risk-taking and coping with the fear that might result. Many definitions of courage [18,19,20] emphasize that courageous actions involve taking risks. Risk comes from the Italian *risco* for “danger”, which means vulnerability to jeopardy. In all sports, athletes often face risks [24]; some put their lives at risk (e.g., extreme sports). While there may be economic risks associated with the sport (e.g., gambling) and social risks (risk of one’s reputation and social status), of central concern, has been the risk of physical injury. A “culture of risks” in sport is primarily characterized by the widespread tolerance of pain and injury [26]. The Venturesome subscale of the SCS-31 include items such as *I risk injury in order not to lose in my sport* and *Even when facing the possibility of injury; I perform to the best of my ability in my sport*. The fifth and final factor of SCS-31 is Self-sacrifice. Courage often involves risking personal harm for a larger goal [11,18]. The Self-Sacrifice subscale of the SCS-31 includes items such as *I do not hesitate to compete, even when facing the possibility of defeat in my sport and I defend my beliefs until the end, even if this action could prove harmful to me in my sport*.

Konter and Toros [27] used the SCS-31 to measure courage in soccer at varying levels and age groups. The results indicated the following: (1) Professionals scored higher than amateurs (*p* < 0.05) and non-substitutes (starters) have higher points than substitutes (*p* < 0.02) related to the Mastery and total score of the SCS-31. (2) Non-substitutes have higher scores (*p* < 0.001) in terms of Determination and Assertiveness. (3) Team captains have higher points (*p* < 0.001) than non-team captains as regard with Assertiveness. (4) Courage scores and the age groups also revealed meaningful differences related to Venturesome and total scores. For example: (a) Players under-17 had higher Venturesome scores than players under-13, (b) Players under-14 had higher Venturesome scores than players under-13, (c) Players under-14 had higher total scores than players under-13. Age under-13 and above-13 of soccer players seem to be critically related to Venturesome.

The present study involved adaptation of the psychometrically sound measurement of the SCS-31 from Turkish into American English. Although the majority of research on courage has been conducted in Western countries [17,28], where the measurement of courage was primarily in English, the SCS-31 was developed in Turkish [4], so its applicability in other cultural groups (e.g., United States) is questionable due to the differences in language, culture, and the perception of courage. Therefore, the present study aims to adapt and validate an American English version of the SCS-31 (SCS-AE), to assess the courage of competitors and athletes within American competitive activities and sporting context.

## 2. Materials and Methods

### 2.1. Participants, Sampling Method and Data Collection

The data collection began after the human research ethical approval from the host university (IRB2012-361 from Clemson University). The research was conducted in accordance with the guidelines set by the International Declaration of Helsinki. In this study, a cross-sectional approach was employed, and a convenience sampling method was applied. Undergraduate students (N = 685) at a mid-sized university in the Southeastern United States completed a survey online for their course or for extra credit. Of these 685 respondents, given the sport-context specific to this courage scale, only 548 athletes who had competition experience in representing the university comprised the final data set for analysis. Participants were on average 18.94 years old (*SD* = 1.21). Approximately half of participants were male (*n* = 268, 48.91%) and half were female (*n* = 274, 50.00%), with six participants not reporting gender (1.09%). The most frequently reported competitive sports were athletics, baseball, basketball, cheerleading, football, golf, lacrosse, soccer, swimming, tennis, and volleyball.

### 2.2. Questionnaire Translation

The first author translated instructions and all items from the original SCS-31 [4] from Turkish to English using Brislin [29] back-translation technique, using an iterative process by independent forward- and back-translation via two professional bilingual translators. A third bilingual translator then back-translated the material from the English back to Turkish. The two versions were compared for equivalence, and the consensus was resolved with the first author. Then, the last authors, together with two native American English speakers, verified the comprehensibility of the translated materials and rechecked for language, cultural relevance and the perception of courage in the local American context.

### 2.3. Measures

The SCS is a relatively recently developed instrument to assess athletes’ courage level. The initial scale consisted of 50 items with five factors. The factors include mastery, determination, assertiveness, venturesome, and self-sacrificial behavior. The items were scored using a five-point Likert-scale from 1 (strongly disagree) to 5 (strongly agree). Konter and Ng [4] have conducted the validation on the 31 items SCS of the Turkish version. They reported 31 items and five factors that fit their data well. The fit indices reported were: χ2 (429) = 584.32, *p* < 0.01, CFI = 0.93, TLI = 0.93, RMSEA = 0.03, SRMR = 0.06 [4]. Internal consistency measured by Cronbach alpha for each subscale ranged from 0.61 to 0.82. The SCS-AE with 31 items was used in the present study.

### 2.4. Statistical Analysis

SCS-AE was analysed by CFA using LISREL version 9.1 and SPSS software (IBM Corp. Released 2017. IBM SPSS Statistics for Windows, Version 25.0. Armonk, NY: IBM Corp). Multiple fit indices were used to determine the fitness of the measurement model on the sample data. The fit indices that were used in this study and the recommended fit values were: the comparative fit index (CFI) and Tucker and Lewis index (TLI) with the desired value of more than 0.95, the root mean square error of approximation (RMSEA) with the desired value of less than 0.07, the root mean square residual (RMR) and the standardised root mean square residual (SRMR) with the desired value of less than 0.08 [30,31]. Other fit indices that commonly presented are the Normed Fit Index (NFI) and the Non-Normed Fit Index (NNFI) with cut-off values of more than 0.90 and 0.95, respectively [30,31]. Items with factor loading less than 0.40 were investigated and removed from the measurement model iteratively after adequate theoretical support was carried out by researchers.

To assess the convergent validity, composite reliability (CR) based on Raykov’s method [32] and average variance extracted (AVE) were computed. The recommended value of CR is 0.70 and above [30] and AVE is 0.50 and above [30]. Cronbach alpha was also reported to assess the internal consistency of the factors of SCS-AE. A Cronbach alpha value of more than 0.60 was considered adequate [33]. Discriminant validity was checked by examining the correlation between the factors in the final best fit model. If the correlation value is not too high and less than 0.85, discriminant validity can be considered achieved [34]. Another alternate test of discriminant validity is that the AVE for each factor is compared with the squared correlations associated with that factor [33].

## 3. Results

### 3.1. Assumption Checking of CFA (Normality of Data Distribution)

Normality of data distribution of the 31 items was examined prior to the CFA in LISREL. Table 1 shows the descriptive statistics, skewness and kurtosis values for all the 31 items. The assumption of multivariate normality for the initial CFA model (31 items) was also checked based on Mardia multivariate skewness and kurtosis tests. The p-values for both tests were significant (<0.05), which indicated that the assumption of multivariate normality of the CFA model was not met. Thus, the estimator MLR, which is robust to non-normality, was used for the subsequent CFA analysis.

### 3.2. Measurement Model of SCS-AE

In the first measurement model (Model 1), 31 items of SCS-31 were included in the CFA. The fit indices for Model 1 indicated that the model fit the data (see Table 2). However, four items had poor factor loading with value below 0.40 (see Table 2). The items were investigated and subjected for removal from Model 1. Model 2 consisted of 27 items (without item D12, M11, M27 and item S15) and the fit indices indicated that model fit the data well. After removal of the four items, the Cronbach’s alpha values for factor determination, mastery, and sacrifice behavior had dropped to 0.77, 0.73, and 0.54, respectively. Model 3 was tested by removing factor sacrifice behavior from Model 2. The fit indices for Model 3 were within the acceptable range, the factor loadings ranging from 0.40 to 0.81.

Table 3 shows the standardized factor loading and Cronbach’s alpha for the three measurement models of SCS-AE with 31, 27 and 24 items. The Cronbach alpha values for all factors in Model 1 and 3 were above 0.60, with acceptable reliability.

Table 4 shows the SCS-AE correlation values for the final measurement model (Model 3) with 24 items, ranging from 0.39 to 0.70. Correlation values were below 0.85, which support discriminatory validity of the construct. However, AVEs for assertiveness and determination are lower than the corresponding inter-construct squared correlation estimates as shown in Table 4. This suggested that, this time, discriminant validity between assertiveness and determination was not supported.

Table 4 shows the CR values for all the factors ranging from 0.69 to 0.77. The CR values exceed 0.70 indicating adequate reliability for the 24 items’ SCS-AE. The AVE of each factor ranged from 0.24 to 0.42. Although the AVE values were below the recommended value of 0.50, the CR were above the recommended value of 0.60, thus the convergent validity of the construct was still considered adequate [35]. Moreover, all factors’ loadings were above 0.40 and model fits reasonably well. Thus, these results support the convergent validity of the measurement model of SCS-AE.

## 4. Discussion

Compared to the original Turkish sample, the present sample replicated three of the five factors from the original scale: Mastery, Determination, and Assertiveness. While not every item remained in our final scale, the basic factor structure of these three subscales was preserved. This suggests that these three constructs may translate well between the two cultures, and, perhaps, might even represent more universal dimensions of courage in sport. By contrast, Venturesome and Self-Sacrifice, two separate factors in Konter and Ng’s [4] Turkish sample, loaded onto only a single factor in the current US sample. These two constructs may be more culturally specific, perhaps due to differences between the US and Turkey in individualism and collectivism [36,37,38]. For example, Konter [38] researched the leadership power perceptions of soccer players and coaches comparing the US and Turkey, and found a four-factor model for Turkey instead of a five-factor model for the US (with the elimination of some items). He concluded that that there seemed to be some degree of cultural convergence and divergence between the two multicultural countries (US and Turkey). Therefore, additional new items and models may be needed for soccer in Turkey. Hofstede [39] has suggested that cultural differences in thought and social action include the factors of power distance, uncertainty avoidance, individualism versus collectivism, masculinity versus femininity, and long-term versus short-term orientation in different cultures. He further argued that people carry mental programs developed in their early childhood in the family and reinforced in schools and organizations, and that these mental programmes contain the national culture component. Cultural differences therefore provide fertile ground for incomprehension and conflict [36,37]. Gauvin et al. [40] indicated the potential for major distortions and inaccuracies in the interpretation of the tests. Besides the apparent language differences, even when the test has been translated accurately, more subtle cultural differences can affect test results. For example, the concepts, relationships and test results valid in Asia or Africa may not apply in North America or Europe, and vice versa [41,42,43].

The first two factors found in Konter and Ng’s [4] Turkish sample seem to address separate functional pieces of Rate’s [44] implicit theory of courage, comprised of voluntary action, a risk to the actor, and pursuit of a good or noble goal. Both address ways in which voluntary action may be enhanced. Mastery, or the ability to face a threat with minimal disruption from emotional upset, may be particularly important in ensuring that the risk does not deter action. Determination, or the ability to focus on goals and persist in achieving them, fits with the goal of courageous action described by Rate [44]. These two factors and the items in them might be relevant to all types of courageous behavior beyond sports.

Finally, the remaining two factors appear to be more specific to sports. The Venturesome–Self Sacrifice items describe being willing to face physical danger or loss in order to compete. The Assertive items describe a particular type of behavior beneficial in sports, assertiveness despite risky conditions. We expect that these two factors may be more particular to sports, motor skills and competitive activities rather than to other areas in which courageous behavior may play out. The general willingness to fight for something important might also be more important for athletes in Turkey compared to the US: compared to the US’s general approval of sports participation. SCS-AE seems to be a promising measure for sports-related courage in US samples. The factor structure of both the SCS-31 and the SCS-AE indicate that courage in sport and competitive activities appear to be characterized by traits related to a variety of courageous action as well as to those specific to situations one might face in sports or competition.

Future research should examine the replicability of these factor structures in both older and younger populations, as well as among those with different educational backgrounds. The extent to which these factors are applicable to a variety of sports is also unknown, although, based on the content of the questions, we might assume that the Mastery and Determination factors may be likely relevant to a wider variety of sports and other competitive activities than the other factors, which appear to be much more relevant for physically risky sports. A variety of related constructs, including risk perception, emotional response to risk, the presence of available knowledge and previous experiences, motivation for sports participation in general, and sports performance, should also be investigated.

## 5. Conclusions

The current study extends Konter and Ng’s [4] scale to a US sample. Together, they provide the start of standardized measurement of courage in sports that can be related to health, performance enhancement, skill development, success, and even continued participation in competitive activities and sports. Overall, the findings suggest supporting the initial efforts to develop SCS-AE. This research showed initial evidence supporting the validity and reliability of the revised version with 18 items and a combined factor of Venturesome and Self-Sacrifice. Using a series of methods, SCS-AE emerged with four factors, namely Assertiveness, Determination, Mastery, and Venturesome–Self-Sacrifice Behavior.

## Figures and Tables

**Table 1:** Descriptive statistics, skewness and kurtosis for the SCS-American English (SCS-AE) 31 items.

Item	Mean	*SD*	Skewness	Kurtosis	Item	Mean	*SD*	Skewness	Kurtosis
M1	2.51	1.05	0.65	−0.56	D17	2.33	0.89	0.75	0.24
D2	2.27	0.99	0.71	−0.21	A18	2.10	0.73	0.52	0.39
A3	2.48	0.81	0.32	−0.24	V19	2.11	0.84	0.61	−0.01
V4	2.48	1.12	0.52	−0.66	D20	2.16	0.88	0.71	0.32
S5	2.04	0.94	0.89	0.34	M21	3.41	1.12	−0.30	−0.93
M6	3.80	0.95	−0.82	0.37	D22	2.44	0.98	0.39	−0.48
D7	1.98	0.91	0.93	0.57	A23	2.34	0.83	0.51	0.26
A8	2.04	0.79	0.70	0.54	M24	3.40	1.01	−0.40	−0.64
V9	2.80	1.04	−0.02	−0.78	D25	1.95	1.95	0.92	1.83
S10	2.23	0.96	0.51	−0.36	A26	2.50	0.95	0.42	−0.43
M11	2.96	0.96	−0.18	−0.86	M27	3.14	0.96	−0.19	−0.61
D12	3.12	0.88	−0.21	−0.78	D28	2.27	0.83	0.70	0.41
A13	2.50	0.89	0.43	−0.51	A29	2.28	0.80	0.48	0.41
V14	2.52	0.99	0.34	−0.79	D30	1.97	0.79	0.91	1.11
S15	2.17	0.87	0.79	0.35	S31	2.56	0.96	0.23	−0.61
M16	3.42	1.03	−0.33	−0.85					

*SD* = Standard deviation, M.

**Table 2:** Value of Goodness-of-fit indices for first measurement model of SCS-AE.

Model	RMSEA	CFI	NFI	NNFI	RMR	SRMR
Model 1 (31 items)	0.06	0.95	0.93	0.95	0.06	0.06
Model 2 (27 items)	0.06	0.96	0.94	0.95	0.05	0.06
Model 3 (24 items)	0.06	0.97	0.96	0.97	0.05	0.06

**Table 3:** Standardised factor loading, cronbach’s alpha of the final measurement model of SCS-AE, 31 items.

Factors/Items	Standardised Factor Loading			Cronbach’s Alpha		
	Model 1	Model 2	Model 3	Model 1	Model 2	Model 3
Assertiveness				0.78	0.78	0.78
A3	0.46	0.46	0.45			
A8	0.48	0.48	0.48			
A13	0.52	0.51	0.51			
A18	0.41	0.41	0.41			
A23	0.52	0.52	0.52			
A26	0.63	0.63	0.63			
A29	0.43	0.43	0.43			
Determination				0.82	0.77	0.77
D2	0.47	0.46	0.45			
D7	0.39	0.40	0.40			
D12	−0.26	-	-			
D17	0.48	0.48	0.48			
D20	0.50	0.50	0.51			
D22	0.58	0.58	0.58			
D25	0.47	0.47	0.48			
D28	0.51	0.51	0.52			
D30	0.46	0.46	0.46			
Mastery				0.82	0.73	0.73
M1	0.40	0.40	0.40			
M6	0.56	0.56	0.56			
M11	0.37	-	-			
M16	0.73	0.73	0.73			
M21	0.80	0.81	0.81			
M24	0.63	0.62	0.62			
M27	0.36	-	-			
Sacrifice behavior				0.61	0.54	-
S5	0.63	0.65	-			
S10	0.39	0.41	-			
S15	0.28	-	-			
S31	0.46	0.49	-			
Venturesome				0.74	0.74	0.74
V4	0.74	0.74	0.73			
V9	0.52	0.52	0.51			
V14	0.73	0.73	0.73			
V19	0.57	0.57	0.58			

**Table 4:** Composite reliability (CR), average variance extracted (AVE), and squared correlations.

Factors	CR	AVE	Factors			
			1	2	3	4
(1) Assertiveness	0.69	0.25	1	0.62	0.19	0.41
(2) Determination	0.71	0.24	0.79 *	1	0.24	0.32
(3) Mastery	0.77	0.41	0.44 *	0.49 *	1	0.07
(4) Venturesome	0.74	0.42	0.65 *	0.57 *	0.26 *	1

Values below diagonal are correlation estimates, * *p*-value < 0.005, values above diagonal are squared correlations.
